# Galectin-1 from cancer-associated fibroblasts induces epithelial–mesenchymal transition through β1 integrin-mediated upregulation of Gli1 in gastric cancer

**DOI:** 10.1186/s13046-016-0449-1

**Published:** 2016-11-11

**Authors:** Yang Chong, Dong Tang, Qingquan Xiong, Xuetong Jiang, Chuanqi Xu, Yuqin Huang, Jie Wang, Huaicheng Zhou, Youquan Shi, Xiaoqing Wu, Daorong Wang

**Affiliations:** Department of Gastrointestinal Surgery, Clinical Medical College of Yangzhou University (Subei People’s Hospital of Jiangsu Province), P.O.BOX: 225001, No.98 Nantong West, Yangzhou, China

**Keywords:** Galectin-1, Cancer-associated fibroblasts, Epithelial–mesenchymal transition, Glioma-associated oncogene 1, β1 integrin, Hedgehog, Gastric cancer

## Abstract

**Background:**

Gastric cancer (GC) is characterized by the excessive deposition of extracellular matrix, which is thought to contribute to this tumor’s malignant behavior. Epithelial-mesenchymal transition (EMT) is regarded as a crucial contributing factor to cancer progression. Galectin-1 (Gal-1), a β-galactoside-binding protein abundantly expressed in activated cancer-associated fibroblasts (CAFs), has been reported to be involved in GC progression and metastasis by binding to β1 integrin, which, in turn, can bind to matrix proteins and activate intracellular cascades that mediate EMT. Increasing evidence suggests that abnormal activation of the hedgehog (Hh) signaling pathway enhances GC cell migration and invasion. The purpose of our study is to explore the role of Gal-1 in the GC progression and metastasis as well as the regulatory mechanism.

**Methods:**

We hypothesized that Gal-1 binding to β1 integrin would lead to paracrine signaling between CAFs and GC cells, mediating EMT by upregulating Gli1. Invasion and metastasis effects of the Gal-1 and Gli1 were evaluated using wound healing and invasion assay following transfection with mimics. Additionally, to facilitate the delineation of the role of the Hh signaling in GC, we monitored the expression level of associated proteins. We also evaluated the effects of β1 integrin on these processes. Furthermore, Gal-1 and Gli1 expression in GC patient samples were examined by immunohistochemistry and western blot to determine the correlation between their expression and clinicopathologic characteristics. The Kaplan-Meier method and Cox proportional hazards model were used to analyze the relationship of expression with clinical outcomes.

**Results:**

Gal-1 was found to induce EMT, GC cell migration and invasion. Further data showed that Gal-1 up-regulated Gli1 expression. β1 integrin was responsible for Gal-1-induced Gli1 expression and EMT. In clinical GC tissue, it confirmed a positive relationship between Gal-1 and Gli1 expression. Importantly, their high expression is correlated to poor prognosis.

**Conclusion:**

Gal-1 from CAFs binds to a carbohydrate structure in β1 integrin and plays an important role in the development of GC by inducing GC metastasis and EMT through targeting Gli1. This study highlights the potential therapeutic value of Gal-1 for suppression of GC metastasis.

**Electronic supplementary material:**

The online version of this article (doi:10.1186/s13046-016-0449-1) contains supplementary material, which is available to authorized users.

## Background

The development of gastric cancer (GC) is a complex, multi-step process associated with an enormous number of genetic alterations, upregulation of cancer-causing genes, downregulation of tumor-suppressor genes, and the acquisition of metastatic ability [1]. A high proportion of deaths related to GC is caused by tumor metastasis, postoperative recurrence, and delayed detection of advanced stage disease [2, 3]. During the process of metastasis and invasion, tumor cells are able to induce a series of changes characterized by diffusible growth factors and cytokines, components of the extracellular matrix, fibroblasts, and myofibroblasts (also called cancer-associated fibroblasts [CAFs]), comprising the tumor microenvironment [4]. Evidence from clinical and epidemiological studies has shown a strong association between CAFs and poor prognosis in several types of cancer, including GC [5, 6].

Metastatic progression, the spread of primary tumors to distant organs, is a complex, multistep physiological process [1]. A large number of studies have shown that epithelial-mesenchymal transition (EMT) plays a critical role in cancer cell invasion and metastasis, and leads to downregulation of epithelial-associated markers such as E-cadherin and upregulation of mesenchymal markers such as vimentin [7]. As a result of EMT, tumor cells acquire metastatic and invasive properties, exhibit characteristics that resemble embryonic mesenchymal cells, and have an enhanced ability to penetrate the surrounding stroma to initiate the formation of new neoplastic foci [8].

Galectin-1 (Gal-1), the first protein of the galectin family of carbohydrate-binding lectins, can act as a biological modifier in tumor growth and metastasis [9]. Our previous work demonstrated that Gal-1, a kind of secretory protein, is overexpressed in CAFs [6], promoting gastric tumorigenesis and angiogenesis [6, 10]. Recently, Bacigalupo *et al.* demonstrated that Gal-1 could induce EMT in hepatocellular carcinoma [11]. However, investigations of Gal-1 in GC are scarce. The expression pattern of Gal-1 in human GC tissues and cell lines, its biological roles, and potential mechanisms contributing to GC progression still need to be addressed.

Major signaling pathways involved in EMT include the TGF-β, Wnt, Notch, and Hedgehog (Hh) pathways [12]. In GC, tumorigenicity and EMT are regulated through activation of glioma-associated oncogene 1 (Gli1), a key member of the Hh signaling pathway [13–15]. Hh signaling during embryogenesis promotes tissue growth, tissue patterning, vascularization, and differentiation, all of which are processes that tumor cells employ in order to support tumor growth and facilitate metastasis [11]. The key signaling molecules in the Hh complex network are considered to be oncogenes, including sonic hedgehog (SHH), smoothened (SMO) and Gli1 [16]. Aberrant activation of Hh/Gli1 has been recognized as a key mediator of metastasis via EMT [17]. Furthermore, dysregulation of Gli1, a key transcription factor in the Hh pathway, has also been shown to affect EMT in cancer cell lines and lymphatic metastasis [16], including GC [18, 19].

The β1 integrin chain (β1 integrin), a member of the integrin family of cellular surface adhesion receptors, contains the lactosamine sequence in its extracellular domain [20]. Overexpression of β1 integrin contributes to a loss of cell adhesion. Gal-1 exerts its biological effects by binding to lactosamine sequences of β1 integrin [20, 21]. He *et al.* have shown that Gal-1 expressed in CAFs promotes the invasiveness of GC cells by direct binding to β1 integrin on the cell surface [22].

Herein, we examined the ability of Gal-1 binding to the carbohydrate structure of β1 integrin to induce EMT via the Hh pathway in GC cell lines.

## Methods

### Patient selection and tissue preparation

From January 2012 to August 2012, 111 patients with GC were treated at the Department of Gastrointestinal Surgery, Clinical Medical College of Yangzhou University (Subei People’s Hospital of Jiangsu Province). The clinicopathological features of these patients are shown in Table 1 and Additional file 1. All patients underwent radical resection; no patients received either chemotherapy or radiotherapy before surgery. This study followed the tenets of the Declaration of Helsinki, and informed written consent was obtained from all patients and controls after clinicians explained the nature and possible consequences of the study. The study protocol was approved by the Medical Ethics Committee of First Clinic Medical School of Yangzhou University (YZU-EC-JS2352).

**Table 1:** Association of Gal-1 and Gli1 with clinicopathological indicators

Parameter	n	Gal-1			Gli1		
		High	Low	*P*-value	High	Low	*P*-value
Tumor size							
< 5 cm	62	30	32	0.118	27	35	0.039
≥ 5 cm	49	31	18		31	18	
Depth of tumor invasion							
T1-T2	31	9	22	0.001	8	23	0.001
T3-T4	80	52	28		50	30	
Histologic type							
Well and moderately differentiated	70	39	31	0.834	37	33	0.868
Poorly and undifferentiated	41	22	19		21	20	
TNM stage							
I- II	30	10	20	0.005	9	21	0.004
III- IV	81	51	30		49	32	
Lymph Nodes Metastasis							
No	43	18	25	0.027	15	28	0.004
Yes	68	43	25		43	25	

### Antibodies

Anti-galectin-1 antibody (Santa Cruz Biotechnology, Santa Cruz, CA, USA), anti-β1-integrin subunit antibody (Cell Signaling Technology, Danvers, MA, USA), anti-SMO antibody (Abcam, Cambridge, UK), anti-Gli1 antibody (Abcam), anti-SHH antibody (Abcam), anti-E-Cadherin antibody (Cell Signaling Technology, Danvers, MA, USA), anti-Vimentin (Cell Signaling Technology), anti-Snail (Cell Signaling Technology), anti-MMP9 (Cell Signaling Technology), anti-β-actin antibody (Beyotime, Jiangsu, China), HRP-conjugated goat anti-mouse IgG and HRP-conjugated goat anti-rabbit IgG (Santa Cruz Biotechnology) were used in this study.

### Immunohistochemistry

Immunohistochemical staining of human paraffin-embedded GC and normal tissue sections was carried out as previously described with minor modifications [10]. Briefly, after antigen retrieval, slides were incubated with primary antibodies against galectin-1 or Gli1 overnight at 4 °C, followed by incubation with biotin-conjugated secondary antibodies, then horseradish peroxidase-conjugated streptavidin. The sections were stained with DAB and counterstained with hematoxylin. Negative controls were treated identically, though the primary antibodies were omitted. Staining density was scored using standard methods, as described previously [10]: negative staining was defined as negative (no visible staining) or weak staining (light brown staining in < 20 % of tumor cells); positive staining as moderate or strong staining (brown or dark brown staining in > 20 % of tumor cells).

### Cell preparations

CAFs were isolated from human gastric carcinoma tissue, while primary human normal gastric fibroblasts (NGFs) were obtained from a non-cancerous region at least 5 cm from the outer tumor margin in the same patient. The primary cells were cultured as our previously described [6]. The human GC lines MGC803, AGS, MNK-45, MKN-28, SGC7901 and BGC-823 were purchased from the Type Culture Collection of the Chinese Academy of Sciences (Shanghai, China). MGC-803 and AGS cells were maintained in Dulbecco’s modified Eagle’s medium (DMEM; Hyclone, Logan, Utah, USA) supplemented with 10 % FBS and the other cell lines including NGFs and CAFs were cultured in Roswell Park Memorial Institute (RPMI) 1640 medium (Hyclone) containing 10 % FBS. All cells were maintained at 37 °C in a humidified atmosphere containing 5 % CO2. Before experiments, GC cells in log phase growth in six-well plates were cultured in media containing only 1 % FBS for 24 h.

### Lentiviral production and transduction

Human *LGALS1* (GenBank accession numberNM_002305) was inserted into the GV248 and GV358 lentiviral vectors (Genechem, Shanghai, China) to silence and upregulate the expression of Gal-1, respectively. The three shRNA sequences were as follows: Gal-1 sh1 (5′-CCGGCACCATCGTGTGCAACAGCAACTCGAGTTGCTGTTGCACACGATGGTGTTTTTG-3′); Gal-1 sh2 (5′-CCGGCCAGCCTGGAAGTGTTGCAGACTCGAGTCTGCAACACTTCCAGGCTGGTTTTTG-3′); Gal-1 sh3 (5′-CCGGGCTGCCAGATGGATACGAATTCTCGAGAATTCGTATCCATCTGGCAGCTTTTTG -3′). GV248 and GV358 lentiviral vectors were constructed to silence and upregulate the expression of LGALS1; a negative control vector containing the cytomegalovirus (CMV) promoter and expressing high levels of green fluorescent protein (GFP) was also created. The negative control was made from GV248 and GV358 too. The lentiviral vectors were transfected into MGC-803 cells at a multiplicity of infection (MOI) ranging from 1 to 100 in the presence of 5 μg/ml polybrene (Sigma-Aldrich, St. Louis, MO, USA). To produce stably transfected cell lines, the cells were cultured in the presence of puromycin (Sigma-Aldrich). The cells were used for subsequent experiments after the expression of the target gene was confirmed using Western blot. After viral infection, the groups were as follows: Gal-1-downregulated CAFs (Down), and gal-1 overexpression CAFs (Over). And Conditioned medium (CM) from NGFs (CM-NGFs), CAFs(CM-CAFs), Over(CM-Over), Down (CM-Down) was obtained by 48 h serum-starved cells, clarified by centrifugation and used freshly.

### Western blot analyses

Total lysates of treated cells were prepared using RIPA buffer containing 1× Tris-buffered saline, 1 % Nonidet P-40, 0.5 % sodium deoxycholate and 0.1 % sodium dodecyl sulfate. Total proteins (50 μg) from each lysate were separated by SDS/PAGE and transferred onto PVDF membranes, and then probed with the indicated antibodies using standard protocols.

### RNA extraction and real-time PCR

Real-time PCR was performed to determine the mRNA expression levels of *LGALS1, SMO, Gli1,* E-cadherin and vimentin. Total RNA was extracted using TRIzol reagent (Invitrogen, Carlsbad, CA, USA) following the manufacturer’s instruction. First-strand reverse transcription was performed using the PrimeScript RT reagent Kit (TaKaRa, Dalian, China). The real-time PCR analyses were conducted on an iQ5 Multicolor Real-Time PCR Detection System (Bio-Rad, Hercules, CA, USA) using SYBR Green Real-time PCR Master Mix (TaKaRa). The PCR program was 30 s at 95 °C followed by 40 cycles at 95 °C for 5 s, 60 °C for 30 s and 72 °C for 30 s. Glyceraldehyde phosphate dehydrogenase (*GAPDH*) was used as the reference control. Fold changes in the mRNA levels of target genes were calculated relative to *GAPDH*. All results are reported as the average ratios of three different independent experiments. The following primers were used: *Gal-1* (forward): CTGGAAGTGTTGCAGAGGTGT and (reverse) CTGGCTGATTTCAGTCAAAGG; *β1-integrin* (forward): TGATTGGCTGGAGGAATGTTA and (reverse) GTTTCTGGACAAGGTGAGCAA; SHH (forward): GACTCAGAGGTGTAAGGACAAGTT and (reverse) CTCGGTCACCCGCAGTTT; SMO (forward) CAGGTGGATGGGGACTCTGTGAGT and (reverse) GAGTCATGACTCCTCGGATGAGG); *Gli1* (forward) GGGATGATCCCACATCCTCAGTC and (reverse) CTGGAGCAGCCCCCCCAGT; N-cadherin (forward) GGCAGAAGAGAGACTGGGTC and (reverse): GAGGCTGGTCAGCTCCTGGC; E-cadherin (forward) TCGTCACCACAAATCCAGTG and (5everse): CATTCACATCAAGCACATCC); vimentin (forward) TGAATACCAAGACCTGCTCAA) and (reverse) ATCAACCAGAGGGAGTGAATC); snali (forward) CACTGCCACAGGCCGTATC and (reverse): CTTGCCGCACACCTTACAG; MMP-9 (forward) TGGGGGGCAACTCGGC and (reverse): GGAATGATCTCTAAGCCCAG; and GAPDH (forward) TGACTTCAACAGCGACACCCA) and (reverse) CACCCTGTTGCTGTAGCCAAA.

### Quantification of Galectin-1 using Enzyme Linked Immunosorbent Assay (ELISA)

NGFs, CAFs, Over and Down culture supernatants were harvested and stored at −80 °C. The level of Gal-1 was determined using a commercial ELISA kit (Boster, Wuhan, China). The ELISA detection sensitivities was ≥156 pg/mL for Gal-1.

### Proliferation assay

The Cell Counting Kit-8 (CCK-8) assay was used as a qualitative index of cell proliferation. Ten thousand cells were plated in 96-well microplates and cell counts performed using a CCK-8 assay (Dojindo, Beijing, China), according to the manufacturer’s protocol. Briefly, 10 μL of CCK-8 solution was added to each well, and the samples were incubated for 1 h before the absorbance was measured at 450 nm. Experiments were performed in triplicate.

### Wound-healing assay

The cells were grown to 80-90 % confluence in a 6-well plate, and a wound was created using a plastic pipette tip across the cell surface. The remaining cells were washed three times to remove cellular debris and incubated at 37 °C with serum-free medium, CM-NGFs, CM-CAFs, CM-Over, and CM-Down containing 10 μg/mL mitomycin C (to block proliferation). The wounded area was photographed at 0 h and 48 h time points. All experiments were performed in triplicate.

### Matrigel invasion assay

GC cell invasion was assessed using a chamber-based invasion assay. In brief, the upper surface of the 24-well transwell pates (pore size, 8.0 μm; Corning, New York, USA) was coated with 150 mg Matrigel basement membrane (BD Biosciences, San Diego, CA, USA). Cells were cultured in serum-free RPMI 1640 medium overnight then the cells centrifuged at 1,000 rpm for 5 min. The cells were re-suspended in serum-free RPMI 1640 medium (st), CM-NGFs, CM-CAFs, CM-Over, or CM-Down, then cell suspensions (200 μl containing 10,000 cells) were seeded onto the filters in 24-well chambers; 500 μl of complete growth was placed in the lower chambers as a chemoattractant. The cells were allowed to migrate for 18 h at 37 °C. Cells remaining on the upper surface of the membrane were removed using a cotton swab. The filters were fixed with 4 % paraformaldehyde, and the cells were stained with 0.05 % crystal violet solution. The cells that had migrated from the upper to the lower side of the filter were counted under a light microscope in 10 randomly-selected fields at 100× magnification. Tumor cell invasion assays were performed in triplicate.

### Transfection of siRNA

The siRNA against *Gli1, β1-integrin* and a negative control siRNA were purchased from Genechem (Shanghai, China). MGC-803 cells seeded in six-well plates were transfected with control or *Gli1* siRNA using Lipofectamine 2000 (Invitrogen, Carlsbad, CA, USA) according to the manufacturer’s instructions. The cells were harvested for further experiments at 24 h after transfection.

### Immunofluorescence microscopy

Prior to immunofluorescence analysis, cells were fixed for 30 min with 4 % paraformaldehyde and permeabilized with 0.2 % Triton-X-100 for 10 min. The coverslips were incubated with primary antibodies at 1:100 dilutions overnight at 4 °C in a humidified chamber. Following incubation phosphate-buffered solution (PBS) washing steps, the appropriate Alexa Fluor 488-linked secondary antibody (Cell Signaling Technology, Danvers, MA, USA) was applied for 2 h at room temperature. The cells were then rinsed with PBS, counterstained with 4′,6-diamidino-2-phenylindole (DAPI), and images were taken on a fluorescence microscope.

### Statistical analyses

Protein expression levels and clinicopathological features were compared using the λ^2^-test. Other data is presented as the mean ± standard error values. Kaplan–Meier survival curve was used to analyze patients’ overall survival rates, and the differences were compared using the Log-rank test. The correlation was analyzed using Spearman’s test. One-way ANOVA with the Least Significant Difference (LSD) post hoc test was used for multiple comparisons using SPSS version 13.0 software (SPSS, Chicago, USA). *P-*values < 0.05 were considered significant.

## Results

### CAF-derived Gal-1 induces EMT in GC cells via activation of the Hh pathway

**Fig. 1:**
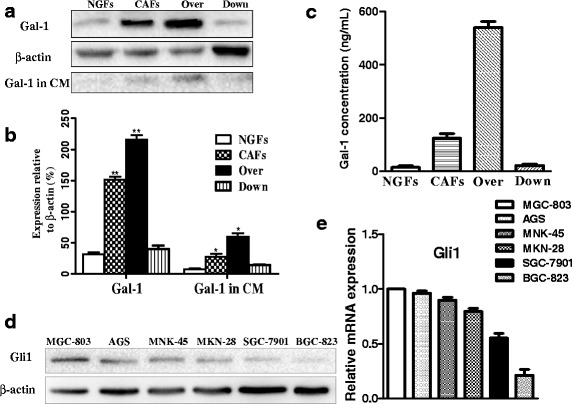
Expression of Gal-1 in CAFs and expression of Gli1 in human GC cells. **a** Western blot showing the expression of Gal-1 in NGFs, CAFs, Over cells, Down cells and their respective conditioned medium (CM). **b** Quantification of Gal-1 expression relative to β-actin. *P < 0.05 vs. NGFs, **P < 0.01 vs. NGFs. **c** Expression of Gal-1 in supernatants was quantified using ELISA. Gal-1 showed increased expression in supernatant from Over cells and reduced expression in supernatant from Down cells. **d** The expression of Gli1 at the protein level in the GC cell lines was determined by western blotting. **e** qRT-PCR analysis of the GC cell lines revealed high levels of Gli1 mRNA in all tested cell lines. Expression levels relative to MGC-803 cells are shown. The expression of each target gene was normalized to the reference gene GAPDH. The bars represent the mean of three independent experiments ± SD

A previous study from our group described the isolation of CAFs and normal gastric fibroblasts (NGFs), and the preparation of recombinant lentiviruses to downregulate Gal-1 expression in CAFs (Down) or overexpress Gal-1 in CAFs (Over) [6]. In the current study, we confirmed that Over cells produced a much larger amount of Gal-1 protein than NGFs (Fig. 1a). Moreover, we confirmed that Gal-1 was strongly expressed in fibroblasts in the stroma of the GC lesion from which CAFs was isolated (Fig. 1a and b). To explore the secretion of Gal-1, the concentration of Gal-1 in the supernatants was quantified by ELISA. Over cells secreted a larger quantity of Gal-1 than CAFs, while NGFs and Down cells did not secrete detectable levels of Gal-1 (Fig. 1c). Gli1 plays a key role in the Hh signaling pathway. We examined the expression of Gli1 in the GC cell lines MGC-803, AGS, MNK-45, MKN-28, SGC-7901 and BGC-823 to identify a cell line for our studies that expressed high levels of Gli1. MGC-803 cells showed the highest level of Gli1 protein and mRNA expression (Fig. 1d and e), and was selected for further in vitro studies.

**Fig. 2:**
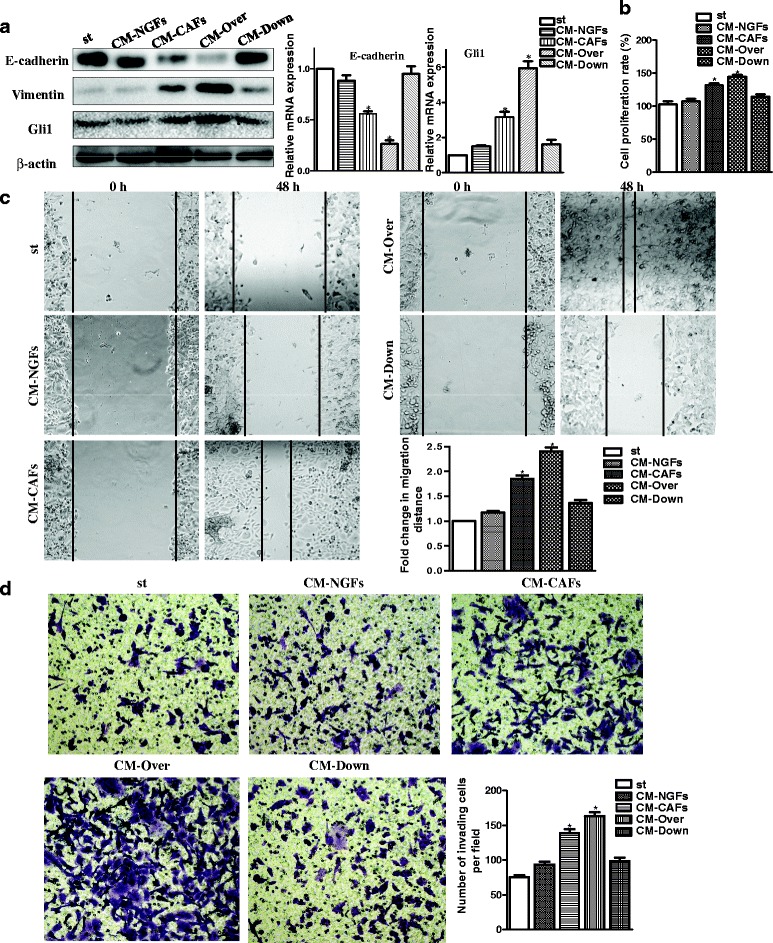
Gal-1 expression affects EMT in GC cells. **a** CM from NGFs (CM-NGFs), CAFs (CM-CAFs), Over cells (CM-Over) and Down cells (CM-Down) was harvested. MGC-803 cells were then treated with serum-free medium (st) or designated CM for 72 h, and the expression of E-cadherin, vimentin and Gli1 monitored by western blot and qRT-PCR. **b** Gal-1 promotes the proliferation of MGC-803 cells. **c** Invasion assay. GC cells treated with CM-Over exhibited significantly enhanced invasion abilities after 18 h in a scratch assay. Bars represent the mean number of invaded GC cells (six fields/sample). *P < 0.05. **d** MGC-803 cells following treatment for 48 h with designated CM (original magnification × 100). *P < 0.05 when compared with st

Interactions between activated fibroblasts in the tumor stroma and tumor cells increase the invasiveness of cancer cells through reducing cell-cell contacts, inducing EMT and promoting metastatic spread [23]. To explore the role of Gal-1 in GC cells, we assessed the expression of E-cadherin in MGC-803 cells following treatment with conditioned media (CM) from NGFs (CM-NGFs), CAFs (CM-CAFs), Over cells (CM-Over) or Down cells (CM-Down). Compared to untreated MGC-803 cells, we found that treatment of MGC-803 cells with CM-Over significantly decreased E-cadherin expression and increased vimentin and Gli1 expression (Fig. 2a). MGC-803 cells were treated with serum free medium, CM-NGFs, CM-CAFs or CM-Down for 48 h. We observed that the proliferation of MGC-803 cells was increased with elevated Gal-1 expression (Fig. 2b). As shown in Fig. 2c and d, MGC-803 cells exhibited significantly enhanced migration and invasion capacity following treatment with CM-Over compared with cells treated with serum free medium, CM-NGFs, CM-CAFs and CM-Down.

**Fig. 3:**
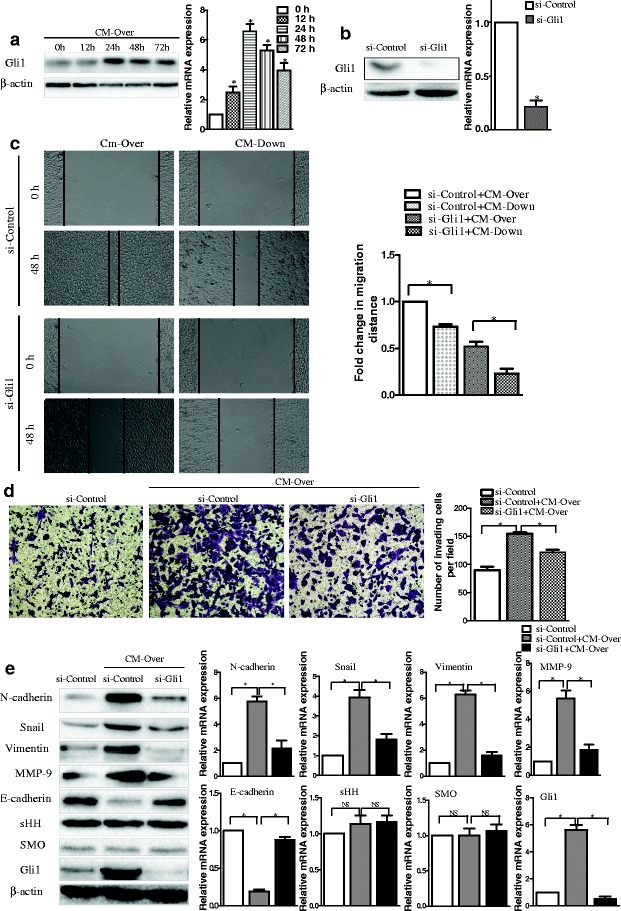
Downregulation of Gli1 abrogates Gal-1-induced migration, invasion and EMT of human GC cells. **a** The expression of Gli1 protein in MGC-803 cells after treatment of Over-CM was evaluated by western blotting (*left*) and qRT-PCR (*right*). Gli1 expression was upregulated in a time dependent manner at both the protein level and the mRNA level. * *P* < 0.05 when compared with expression at 0 h. **b** Silencing of Gli1 expression by siRNA at 48 h following transfection was confirmed by western blotting (*left*) and qRT-PCR (*right*). *P < 0.05 when compared with the si-Control. **c** Gal-1-induced GC cell migratory ability was impaired by Gli1 knockdown. Representative images of GC cells treated for 48 h with CM from Over cells and Down cells exhibited significantly enhanced migratory abilities. These effects were abolished by Gli1 knockdown. *P < 0.05. **d** The effect of Gal-1 on cell invasion was impaired by Gli1 knockdown (original magnification × 100). *P < 0.05 when compared to control cells without treatment with Over-CM. **e** Loss of Gli1 expression reverses the effects of Gal-1-induced EMT in GC cells. *P < 0.05. These results are presented as the mean ± SD of three independent experiments

Although Gal-1 influenced expression of E-cadherin, the mechanisms through which the process occurs remain unclear. The Hh pathway guides cellular proliferation and differentiation in developmental pathways, so its potential for inducing EMT is unsurprising [24]. Consequently, we hypothesized that Gal-1 induces EMT via activation of the Hh signaling pathway. We observed that GC cells exposed to Gal-1 exhibited enhanced migratory and invasive potentials and underwent EMT. To understand the influence of Gal-1 activation on the Hh signaling pathway, we investigated the expression of Gli1 in MGC-803 cells. To our surprise, there was a substantial increase in Gli1 expression at the levels of mRNA and protein when MGC-803 cells were treated with CM-Over (Fig. 3a).

To assess whether the activation of the Hh target gene Gli1 is involved in Gal-1-induced EMT in GC cells, we knocked down Gli1 expression in MGC-803 cells using Gli1 siRNA (Fig. 3b). A wound-healing assay (Fig. 3c), used to measure migration, demonstrated moderate wound closure in Gli1 knockdown cells in the presence of CM-Down compared with other conditions. In addition, a cell invasion assay revealed that the enhanced invasiveness of MGC-803 cells in the presence of CM-Over was abolished by Gli1 knockdown and the number of migratory cells reduced by 50 % compared with control siRNA-treated cells (Fig. 3d). As shown in Fig. 3e, the reduced expression of the epithelial marker E-cadherin in response to CM-Over treatment was partially reversed with Gli1 knockdown. Gal-1-induced expression of snail and the mesenchymal markers N-cadherin and vimentin was greatly reduced compared with cells transfected with control siRNA. These results indicate that Gal-1-induced invasion and EMT of the human GC cells is mediated, at least in part, by Gli1. Consistent with the inhibition of EMT, Gal-1-induced up-regulation of the invasion-related protein MMP-9 was partially abolished by Gli1 knockdown in GC cells (Fig. 3e). These data suggest that activation of the Hh pathway plays a pivotal role in Gal-1-induced GC cells invasion and EMT.

### Activation of the Gal-1/β1 integrin axis induces the expression of Gli1

Integrins in the extracellular matrix (ECM), including β1 integrin, bind with extracellular Gal-1 [9, 20–22, 25, 26]. Integrins appear to play important roles during pregnancy through their involvement in cell invasion, migration and EMT [27]. Previous studies revealed that extracellular Gal-1 secreted by CAFs enhanced the invasiveness of GC cells by upregulating the expression of β1 integrin [22]. Similarly, higher expression of β1 integrin was also shown to induce EMT through the aberrant activation of the Hh pathway [28]. Therefore, we speculated that Gal-1 could activate the Hh pathway via binding to β1 integrin.

**Fig. 4:**
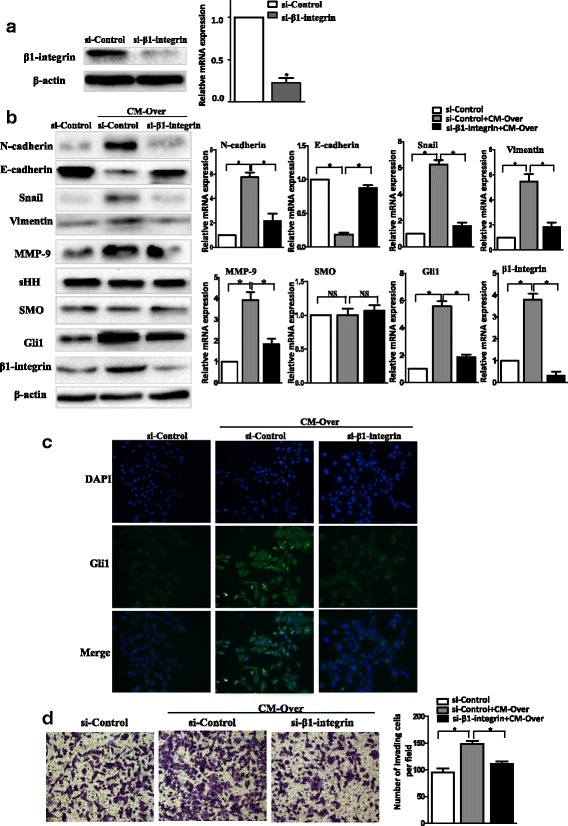
β1 integrin is necessary for Gal-1-induced effects in GC cell migration, invasion, and EMT. **a** The silencing of β1-integrin expression by siRNA at 48 h was confirmed using western blotting (*left*) and qRT-PCR (*right*). *P < 0.05 when compared with the si-Control. **b** The expression of EMT-related molecules (E-cadherin, N-cadherin, vimentin, and snail), invasion-related molecules (MMP-9), key molecules in the Hh pathway (SHH, SMO, and Gli1) and β1 integrin were analyzed by western blotting (*left*). The mRNA expression levels were determined by qRT-PCR (*right*). *P < 0.05. The results are presented as the mean ± SD of three independent experiments. **c** MGC-803 cells were labelled with fluorescein-conjugated Gli1 specific antibody (green) following treatment with CM from Over (magnification × 200). The nuclei were stained with 4′,6-diamidino-2-phenylindole (DAPI). **d** The invasion-promoting effects of Gal-1 were abolished by Gli1 knockdown. The number of invading cells was quantified under a light microscope by counting six random fields at a magnification of 200×. *P < 0.05. The data represent the results of three independent experiments

To confirm whether binding of Gal-1 to β1 integrin promoted activation of the Hh signaling pathway, we knocked down β1 integrin in MGC-803 cells using siRNA (Fig. 4a). We examined the expression of EMT-related molecules in control and Gal-1-stimulated MGC-803 cells using western blotting to measure protein levels and qRT-PCR to measure RNA levels. As shown in Fig. 4b, the expression of E-cadherin was significantly reduced in MGC-803 cells after treated with CM-Over at both protein and mRNA levels (*P* < 0.05). The protein and mRNA expression of N-cadherin, vimentin, and snail were increased dramatically. In contrast, there were no obvious increases in the expression of N-cadherin or vimentin in MGC-803 cells exposed to Gal-1 when β1 integrin was knocked down. The expression of E-cadherin in MGC-803 cells was unaffected compared with control cells cultured in the absence of CM-Over. Up-regulation of Gli1 expression in response to CM-Over was abolished when β1 integrin was knocked down in MGC-803 cells, while the expression of SHH and SMO remained unchanged (Fig. 4b). These results were also confirmed by immunocytochemistry assays; MGC-803 cells showed markedly increased expression of Gli1 when grown in the presence of CM-Over, while the increased expression was abolished by integrin β1 knockdown (Fig. 4c). These findings suggest that β1 integrin activation is essential for the Gal-1-induced activation of the non-canonical Hh pathway in GC cells. As expected, following treatment with CM-Over, MGC-803 cells exhibited enhanced invasive abilities compared with untreated cells (Fig. 4d). MGC-803 cells with β1 integrin knockdown exhibited impaired invasive abilities even when they were exposed to higher expression of Gal-1. Taken together, these data indicate that Gal-1 can induce EMT through stimulating Gli1 expression in GC cells, a process that is mediated by β1 integrin, a receptor of Gal-1.

### Correlation between Gal-1 and Gli1 expression, and clinicopathological features

**Fig. 5:**
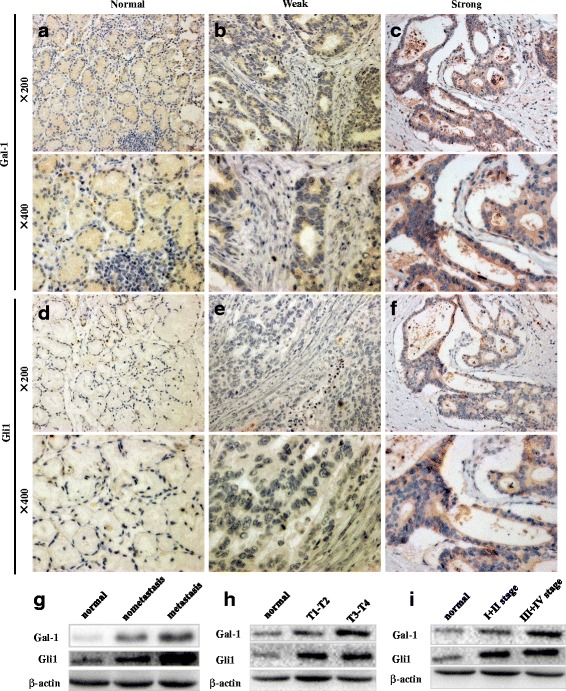
Immunohistochemical staining of Gal-1 and Gli1 in GC sections. Expression of Gal-1 is low in normal tissues (**a**), while staining ranges from weak (**b**) to strong (**c**) in GC tissues. Likewise, Gli1 shows low expression in normal tissues (**d**), while expression ranges from low to high in GC tissues. Western blot analysis of Gal-1 and Gli1 expression in GC tissues showing that expression is significantly correlated with lymph node metastasis (**g**), the depth of tumor invasion (**h**), and TNM stage (**i**)

We performed immunohistochemical analysis to evaluate the clinical significance of Gal-1 and Gli1 expression in 111 GC tissue samples. Gal-1 showed significantly higher expression in 54.95 % (61/111) of GC tissues compared to adjacent normal tissues (Fig. 5a–c). Likewise, Gli1 expression was detected in 52.25 % (58/111) of GC tissues, indicating that Gli1 expression was higher than in adjacent normal tissues (Fig. 5d–f). Moderate Gal-1 staining was detected in the stroma of normal mucosa, while the Gal-1 staining intensity was significantly higher in the stroma and epithelium of the GC tissues. Gli1 staining was predominantly observed in the cell cytoplasm and nucleus. Spearman’s correlation analysis indicated a positive association between Gal-1 and Gli1 expression in GC tissues (r = 0.875, *P* = 0.000).

As shown in Table 1, Gal-1 expression was highly associated with the depth of tumor invasion (*P* = 0.001), lymph node metastasis (*P* = 0.027), and TNM stage (*P* = 0.005). Meanwhile, Gli1 expression was significantly associated with tumor size (*P* = 0.039), the depth of tumor invasion (*P* = 0.001), lymph node metastasis (*P* = 0.004), and TNM stage (*P* = 0.004). Figure 5g-i, which shows western blots of GC tissues, confirmed the positive correlation between Gal-1 and Gli1 expression.

### Both Gal-1 and Gli1 expression are associated with a poor prognosis in GC patients

**Fig. 6:**
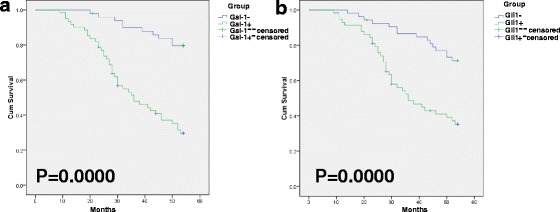
Prognostic significance of Gal-1 and Gli1 expression in GC patients. Kaplan–Meier analysis of overall survival based on Gal-1 (**a**) Gli1 (**b**) expression in 111 patients. Kaplan–Meier survival curves show that high Gal-1 and Gli1 expression is associated with poor overall survival

As shown in Fig. 6a and b, patients were divided into low and high expression groups based on IHC scores. Kaplan–Meier curves and the log-rank test were used to assess the prognostic value of Gal-1 and Gli1 in gastric tumors. The results showed a significant difference in overall survival probability between groups of patients with high expression of Gal-1 and Gli1 and those with low expression of Gal-1 and Gli1. Statistical analyses demonstrated that high Gal-1 and Gli1 expression was associated with poor overall survival (*P* = 0.000 and *P* = 0.000, respectively). Furthermore, in univariate analysis, patients with high expression of Gal-1 and Gli1 had a markedly higher risk of death [HR 0.036 (0.010–0.135), *P* < 0.001; 5.239 (1.554–17.668), *P* < 0.01, respectively, Table 2]. In addition, the remaining indicators, including the depth of tumor invasion, lymph node metastasis and TNM stage, were also significantly correlated with overall survival based on univariate analysis (Table 2). However, as show in Table 2, age, gender, size and differentiation status had no prognostic value. The multivariate analysis further demonstrated that the expression of Gal-1 and Gli1 were independent prognostic factors for GC patients (*P* = 0.000 and *P* = 0.002, respectively; Table 2).

**Table 2:** Univariate and multivariate analysis of prognostic indicators in gastric cancer patients

Indicator	Univariate analysis		Multivariate analysis	
	HR (95 % CI)	*P-*value	HR (95 % CI)	*P--*value
Age (<60/≥ 60)	0.691 (0.373–1.279)	0.239	NA	NA
Gender (male/female)	1.070 (0.586–1.954)	0.826	NA	NA
Tumor size (<5 cm /≥ 5 cm)	1.812 (0.945–3.476)	0.074	NA	NA
Depth of tumor invasion (T3-T4/T1-T2)	8.285 (1.229–55.866)	0.030	6.481 (1.108–37.907)	0.038
Histologic type (well, moderate/poor, undifferentiated)	1.168 (0.646–2.114)	0.607	NA	NA
TNM stage (III- IV /I- II)	0.083 (0.011–0.624)	0.016	0.142 (0.023–0.865)	0.034
Lymph Nodes Metastasis (yes/no)	0.131 (0.044–0.395)	0.000	0.162 (0.058–0.455)	0.001
Gal-1 (+ ~ +++/−)	0.036 (0.010–0.135)	0.000	0.035 (0.009–0.128)	0.000
Gli1 (+ ~ +++/−)	5.239 (1.554–17.668)	0.008	6.401 (1.958–20.928)	0.002

## Discussion

The stroma reaction involves secreted factors from both host and tumor cells that function to promote the desmoplastic response and accelerate the progression of GC, highlighting the complex interplay between cancer cells and the stroma involved in the induction of EMT [29]. Migration and invasion are two basic features of malignant tumor cells. EMT is an important mechanism in tumor invasion and metastasis in which the adhesion protein E-cadherin plays a central role [8, 12]. Gal-1 was reported to induce EMT in hepatocellular carcinoma [11], but no data on GC has been provided to-date. Therefore, we analyzed the connection between Gal-1 and EMT in GC. Our present work demonstrates that Gal-1 secreted by CAFs could play a role in the progression of GC. Elevation of Gal-1 levels in the tumor microenvironment promoted the transition from epithelial cell morphology to the fibroblastoid phenotype, loss of the adherens junction protein E-cadherin and an increase of the mesenchymal marker vimentin.

As one of the most important signaling pathways, Hh is involved in the regulation of GC cell proliferation, migration, invasion, stem cell maintenance, and lymphangiogenesis [30]. Gli1 has been identified as the final transcriptional effector and the key marker of the Hh signaling pathway, and has been shown to be activated in various human malignancies such as prostate cancer and gastrointestinal cancer [30, 31]. Additionally, a previous study showed that, in GC, the levels of the Hh pathway marker Gli1 were associated with levels of the EMT markers E-cadherin and snail [32]. Interestingly, a recent study by Wang *et al.* suggested that Gal-1 is required for Hh signaling pathway activation in pancreatic cancer [33]. In our study, to determine the mechanism of Gal-1-regulated EMT in GC, we modified Gal-1 expression levels and examined the effects on Gli1 expression. As anticipated, increasing the expression of Gal-1 resulted in upregulation of Gli1, further demonstrating the positive association between Gal-1 and Gli1 in GC. Examination of the protein expression levels of EMT markers, as well as migration and invasion assays showed that knock down of Gli1 in MGC-803 cells abolished the effects of Gal-1 in promoting EMT, suggesting that Gli1 plays an important role in Gal-1-induced EMT in GC. Gal-1 may have a variety of roles in tumor metastasis, and our research clearly showed that Gli1 is involved in the promotion of EMT-induced metastasis by Gal-1. We further revealed that overexpression of Gal-1 in CAFs induced cancer cells to achieve a more malignant phenotype through a non-classical Hh signaling pathway. However, the correlation between Gal-1 and Gli1 in GC and their clinicopathological significance has not been reported in the literature. This is the first report revealing a positive relationship between Gal-1 expression and the levels of Gli1, an important member of Hh signaling pathway in vivo, suggesting that the interaction of Gal-1 and the Hh signaling pathway may be implicated in primary GC tumorigenesis, progression, and metastasis. Furthermore, most studies confirm that high Gal-1 [10] and Gli1 [32] expression is significantly associated with worse clinicopathological parameters and decreased overall survival in GC.

Gal-1 is a secreted protein present in the extracellular matrix [34]. Regardless of whether or not the signal peptide is present in the primary polypeptide sequence, Gal-1 exists as a non-covalent homodimer in its secreted form [34]. Moiseeva *et al.* demonstrated that Gal-1 directly binds to β1 integrin but not to the α subunits on the cell surface, enhances activation of β1 integrin, and regulates multiple tumor-promoting functions [21]. Gal-1 binds to lactosamine sequences [20]. The membrane receptor-type protein β1 integrin contains the lactosamine sequence in its extracellular domain [20]. He *et al.* have shown that Gal-1 in CAFs promote the invasiveness of GC cells by direct binding to β1 integrin on the cell surface [22]. Additionally, their work has revealed a positive correlation between Gal-1 and β1 integrin expression in GC [22]. In addition, the downregulation of β1 integrin reduces the expression of Gli1 in human prostate prostate cancer cells [35]. Our study found that GC cells exhibited enhanced invasion as well as up-regulation of Gli1 in response to CM from Over cells. These effects were abolished by β1 integrin knockdown. Furthermore, the ability of Gal-1 to stimulate EMT was reversed by knockdown of β1 integrin. These findings suggest that β1 integrin activates the Hh pathway, resulting in the triggering of Gal-1-mediated EMT in GC.

## Conclusions

Our present results, combined with preliminary findings from other groups, have provided evidence that high expression of Gal-1 in GC tissues contributes tumor cells migration and invasion via Gli1-driven EMT after binding of Gal-1 to β1 integrin. These findings not only improve our understanding of the molecular mechanisms underlying the effects of Gal-1 in GC metastasis, but also provide new insight into Gal-1 as an important therapeutic target associated with GC metastasis.

## Additional file

Additional file 1: Supplementary Table. Association of Gal-1 and Gli1 with clinicopathological indicators: age and gender. (DOC 33 kb)

## Abbreviations

CAFs: Cancer-associated fibroblasts
CM: Conditioned medium
EMT: Epithelial-mesenchymal transition
Gal-1: Galectin-1
GC: Gastric cancer
Gli1: Glioma-associated oncogene 1
Hh: Hedgehog
NGFs: Normal gastric fibroblast cells
SHH: Sonic hedgehog
SM0: Smoothened
