# Supplementary material for: Galectin-1 from cancer-associated fibroblasts induces epithelial–mesenchymal transition through β1 integrin-mediated upregulation of Gli1 in gastric cancer
Source: J Exp Clin Cancer Res. 2016 Nov 11;35:175. doi: 10.1186/s13046-016-0449-1 (PMC5106768; doi:10.1186/s13046-016-0449-1)
Supplement: Additional file 1: — Supplementary Table. Association of Gal-1 and Gli1 with clinicopathological indicators: age and gender. (DOC 33 kb) [file 13046_2016_449_MOESM1_ESM.doc]

| **Supplementary Table** Association of Gal-1 and Gli1 with clinicopathological indicators: Age and Gender | | | | | | | |
| --- | --- | --- | --- | --- | --- | --- | --- |
| Parameter | n | Gal-1 |  |  | Gli1 |  |  |
|  |  | High | Low | *P*-value | High | Low | *P*-value |
| Age (years) |  |  |  |  |  |  |  |
| < 60 | 53 | 32 | 21 | 0.272 | 30 | 23 | 0.380 |
| ≥ 60 | 58 | 29 | 29 |  | 28 | 30 |  |
| Gender |  |  |  |  |  |  |  |
| Male | 76 | 45 | 31 | 0.184 | 44 | 32 | 0.079 |
| Female | 35 | 16 | 19 |  | 14 | 21 |  |
